# Radiomics-based explainable artificial intelligence to predict treatment response following lung stereotactic body radiation therapy

**DOI:** 10.1093/bjr/tqaf043

**Published:** 2025-10-10

**Authors:** Savino Cilla, Carmela Romano, Gabriella Macchia, Donato Pezzulla, Elisabetta Lepre, Milly Buwenge, Costanza Maria Donati, Erika Galietta, Alessio Giuseppe Morganti, Francesco Deodato

**Affiliations:** Medical Physics Unit, Responsible Research Hospital, Campobasso 86100, Italy; Medical Physics Unit, Responsible Research Hospital, Campobasso 86100, Italy; Radiation Oncology Unit, Responsible Research Hospital, Campobasso 86100, Italy; Radiation Oncology Unit, Responsible Research Hospital, Campobasso 86100, Italy; Istituto di Radiologia, Università Cattolica del Sacro Cuore, Roma 00100, Italy; Radiation Oncology Unit, IRCCS Azienda Ospedaliero-Universitaria di Bologna, Bologna 40100, Italy; Radiation Oncology Unit, IRCCS Azienda Ospedaliero-Universitaria di Bologna, Bologna 40100, Italy; Radiation Oncology Unit, IRCCS Azienda Ospedaliero-Universitaria di Bologna, Bologna 40100, Italy; Radiation Oncology Unit, IRCCS Azienda Ospedaliero-Universitaria di Bologna, Bologna 40100, Italy; Department of Medical and Surgical Sciences (DIMEC), Alma Mater Studiorum—Bologna University, Bologna 40100, Italy; Radiation Oncology Unit, Responsible Research Hospital, Campobasso 86100, Italy; Istituto di Radiologia, Università Cattolica del Sacro Cuore, Roma 00100, Italy

**Keywords:** lung metastasis, radiomics, SBRT, SHAP, predictive models

## Abstract

**Objectives:**

To develop and validate a CT-based radiomic-clinical-dosimetric model to assess the treatment response of lung metastasis following stereotactic body radiation therapy (SBRT).

**Methods:**

Eighty lung metastases treated with SBRT curative intent in a single institution were analysed. The treatment responses of lung lesions were categorized as a complete responding (CR) group vs a non-complete responding (NCR) group according to Response Evaluation Criteria in Solid Tumors (RECIST) criteria. For each lesion, 107 features were extracted from the CT planning images. The least absolute shrinkage and selection operator (LASSO) was used for features selection. An eXtreme Gradient Boosting (XGBoost) model was trained and validated. Shapley additive explanations (SHAP) analysis was used to provide insights into the impact of each variable on the model’s predictions.

**Results:**

Eight radiomic features, 1 dosimetric variable, and no clinical variables were identified by LASSO and used to build the XGBoost model. The model yielded areas under the curve (AUCs) of 0.897 (95% CI 0.860-0.935) and 0.864 (95% CI 0.803-0.924) in the training cohort and validation cohort, respectively. Skewness, surface-to-volume ratio, sphericity, and biological equivalent dose (BED10) were the most significant variables in predicting CR. The SHAP plots illustrated the feature’s global and local impact to the model, explaining the model output in a clinician-friendly way.

**Conclusion:**

The integration of the XGBoost model with the SHAP strategy was able to assess lung lesions CR following SBRT, with the potential to assist clinicians in directing personalized SBRT strategies in an understandable manner.

**Advances in knowledge:**

The explainable radiomics model we propose can better predict the treatment response of lung metastasis after SBRT and provide further guidance for clinical practice.

## Introduction

Lung is a common site for metastases for most solid tumours with up to 54% of cancer patients developing lung metastases during their disease.^1^ Unlike patients with aggressive metastatic dissemination, typically treated with systemic therapy, patients with oligometastatic disease or a modest metastatic burden are more likely to benefit from local treatment.^2^ As a potentially beneficial alternative to surgery for local treatment, stereotactic body radiation therapy (SBRT) is currently considered to be on par with other available treatment modalities.^3^ Many studies in the oligometastic scenario have reported local control rates of 70%-100% at 1 year, with prolonged local control associated with smaller tumour sizes and high biological equivalent doses (BEDs) (≥100 Gy).^4–6^

Lung and metastases exhibit significant heterogeneity in terms of associated stroma and vasculature, as well as genomic expression, with potentially significant effects on prognosis.^7^ As a result, the tumour’s clinical features might not fully capture its heterogeneity. This limitation may be partially addressed with the development of radiomics, as several features that are clinically undetectable can be extracted to capture tumour heterogeneity within and between lesions^8–10^ and then associated with the tumours’ biologic structure and the surrounding microenvironment.^11^ While most research in radiomics for lung metastases is still exploratory, some studies have been able to obtain radiomics signatures for the prediction of treatment efficacy, including overall survival^12–14^ and nodal relapse and recurrence rate.^15^^,^^16^ Studies that focused on the efficacy of lung SBRT treatments in terms of treatment response are much rarer.^17^^,^^18^ Owing to the widespread adoption of SBRT for lung oligometastases, there is a strong need of new predictive models, based on radiomics analysis, in order to differentiate responsive and non-responsive tumours to treatment. In other words, in this clinical setting, the paucity of reliable and accurate predictive models is a significant obstacle to the delivery of tailored treatment for lung cancer patients, particularly in view of the recent discoveries of radiomics features as potential prognostic value.

Over the past few years, the rapid advancement in data-driven technologies has influenced also the treatment response modelling and outcomes prediction in radiation oncology. Advanced machine learning (ML) models can incorporate patient-specific radiosensitivity in outcome modelling by leveraging data on patients’ relevant radiogenomics biomarkers both before and during radiation treatment.^19^ However, although a number of ML models have shown promise, they are generally regarded as “black boxes,” that is, they are uninterpretable with respect to the importance of each feature on the output prediction. This is undesirable because clinicians will lose trust as a result of the prediction’s unclear understanding. The term “explainable artificial intelligence” (XAI) refers to the methods and approaches used to build AI systems that enable end-users to understand and interpret the results and predictions produced by AI models. Because of the increasing deployment of “opaque” AI applications, the need for clarity and explainability stems especially from the potential high-impact consequences of erroneous AI predictions in a critical field as healthcare. Therefore, the effective implementation of AI models in clinical routine strongly hinges on their capacity to be explainable and transparent about their decision-making processes and underlying logic. A recent proposed method able to provide global and local explanations for ML models is the Shapley additive explanations (SHAP) values, a post hoc interpretable algorithm that uses additive attribution to convert SHAP values from the ML feature space to the clinical variable space.^20^ This transformation acts within a game-theoretic framework that interprets each feature as a player in a game where a feature’s individual contribution to the final prediction is assessed then allowing to assess the local and global importance of features and improving the interpretability of the model. Moreover, another limitation of ML models includes the imbalance of outcome classes that are responsible for skewed performance, translating to lower sensitivity and higher misdiagnosis rates. A recent strategy able to deal with imbalanced scenarios is at an algorithmic-level approach, where new classifiers such as the eXtreme Gradient Boosting (XGBoost) are adapted to handle imbalanced data by weighting the minority class and changing the sample weight distribution. XGBoost was proposed by Chen et al^21^ as an effective tree-based ensemble ML algorithm, and it has shown higher capability for modelling complex systems in terms of prediction accuracy, interpretability and classification versatility.^22^

To this end, the present study aims to develop an explainable ML framework using radiomics and clinical data by connecting the XGBoost model to the SHAP algorithm to predict the treatment response of lung metastasis following SBRT. In particular, we aim to describe how this explainable ML model can potentially be used in the clinic to identify the most predictive covariates and inform treatment decision-making for patients undergoing SBRT.

## Methods

### Study cohort

Fifty-six patients with 80 lung oligometastases previously treated with SBRT at our hospital were retrospectively evaluated. Patients were part of a prospective Phase I-II trial^23^ approved by the Institutional Review Board of the Catholic University Institutional Review Board (Destroy-1: P#594/CE). Written informed consent was given by each patient.

### CT image acquisition, segmentation, and planning

Patients were simulated in the supine position. CT planning images were obtained with a 128-slice scanner (Brilliance 128, Philips Healthcare, Best, the Netherlands) using 2-mm slice thickness. The clinical target volume (CTV) was identified as the gross tumour volume (GTV). A patient-specific internal target volume (ITV) was defined on the basis of the respiratory excursion’s analysis (free breathing or abdominal compression or deep inspiration breath-hold). Based on a previous analysis of patient’s set-up reproducibility,^23^ the set-up margin (SM) was set at 3 mm for all patients. The planning target volume (PTV) was defined based on the evaluation of ITV and SM.

All patients received a prescribed dose ranging from 30 to 50 Gy in 5 consecutive fractions. Using an α/β ratio of 10, this is equivalent to a BED10 ranging from 60 to 100 Gy. All SBRT plans were generated using 6 MV X-ray energy beams with fixed-field intensity modulated (IMRT) or the volumetric modulated arc therapy (VMAT) techniques. Doses were calculated using the collapsed cone convolution algorithm with a dose grid size of 2 mm. The target coverage was defined as optimal if at least 95% of PTV was irradiated with the 100% of dose prescription and all hot spots (up to 140% of prescription dose) fell within the GTV.

### Treatment response evaluation

The treatment radiological response, as determined by morphological contrast-enhanced CT 4 months after SBRT, was considered as the primary clinical outcome. According to the RECIST (Response Evaluation Criteria in Solid Tumors) system v1.0,^24^ this time was considered suitable for both early and late responder cancer. Complete response (CR) was defined as the disappearance of target lesions on CT-scan; a decrease of more than 30% was classified as a partial response (PR); disease progression or a null response was classified as a non-responder (NR). The occurrence of tumour response was converted into a binary outcome: positive for patients who experienced complete response (CR) and negative for any other eventuality (NCR).

### Radiomics features and analysis

All the features needed for radiomics analysis were extracted from the CT DICOM images containing the GTV segmentation. Image processing was performed with the Pyradiomics 2.0.1 software package,^25^ able to extract standardized features as specified by the Image Biomarker Standardization Initiative (IBSI).^26^ Totally, 107 features (19 first-order statistics features, 26 shape-based histogram features, 16 grey-level co-occurrence matrix features, 16 grey-level size-zone matrix features, 5 neighbouring grey-tone difference matrix features, and 14 grey-level dependence matrix features) were automatically extracted for each lesion. Pre-processing steps involved the resampling the CT images to isotropic voxels of 1 mm^3^ using standard Pyradiomics procedures (B-spline interpolation for the CT images) and the discretization of the voxels’ intensity using a fixed bin of 25 Hounsfield units. Moreover, the manual segmentation of the GTV allowed to remove any voxels with Hounsfield units below −400 HU and above 1000 HU, which were thought to represent normal lung and bony tissue inadvertently included in the GTV. Supplementary material provides a summary of all parameters set in Pyradiomics during the feature extraction.

### Data preparation

The stability of the radiomics features was assessed by evaluating the concordance correlation coefficients (CCCs) between every couples of radiomics features obtained by 2 segmentations carried out by a radiation oncologist and a radiologist. This analysis was performed for 30 randomly selected lesions. In order to avoid overfitting and reduce the dimensionality of variables, the least absolute shrinkage and selection operator (LASSO) regression method was performed to identify potential variables associated with the complete treatment response. This is an embedded method based on the incorporation of an L1-norm regularization term into a linear regression model. It is widely used for feature selection since it shrinks the coefficients of less significant features to zero, selecting a small subset of features to form the final potential descriptor group. In particular, also if there are redundant features (ie, strong correlation between them), LASSO selects 1 and reduces the value of the others to zero.

**Figure 1.**
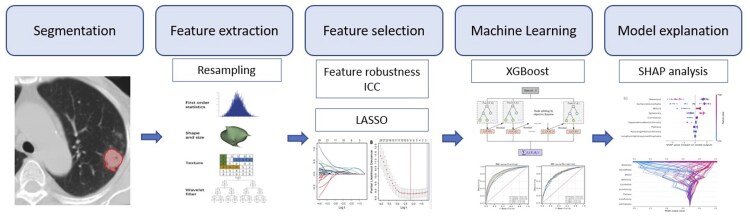
Overall flowchart of the study. Abbreviations: ICC = Intraclass Correlation Coefficient; LASSO = least absolute shrinkage and selection operator; SHAP = Shapley additive explanations; XGBoost = eXtreme Gradient Boosting.

Figure 1 shows an overview of the complete analytical flowchart.

### ML modelling

XGBoost is a recent algorithm, today considered as the state-of-the-art in ML, able to solve the well-known limitation of the traditional ones in handling large datasets, missing values, and complex interactions between features by combining the power of gradient boosting with efficient tree learning algorithms. More in detail, XGBoost is an ensemble learning method that combines the predictions of multiple weak models to produce a strong prediction. Gradient boosting is used to train the decision trees, which are the weak models in XGBoost. This indicates that the algorithm fits a decision tree to the residuals of the previous iteration. The following objective function is used to train the decision trees XGBoost:


$$\min_{\theta }\left(\sum_{1}^{n}l\left(y_{i},y_{i}^{\prime }\right)+\sum_{1}^{K}\Omega \left(f_{k}\right)\right)$$

where *l* is the loss function, *y_i_* is the true label of the *i*_th_ training example, $y_{i}^{\prime }$ is the predicted label of the *i*_th_ training example, *f_k_* is the *k*_th_ decision tree, and Ω is a regularization term that penalizes the complexity of the trees. This objective function is optimized using gradient descent. Following the training of the decision trees, XGBoost generates predictions by averaging the weighted predictions of each tree. Also, during training, the same objective function is used to determine the weights for each tree. As a result, the algorithm is able to determine which trees are more significant and need to receive a greater weight in the final prediction.

The entire dataset was divided into a test and train dataset in a stratified manner using an 80:20 ratio. The fine-tuning of hyperparameters was performed using the GridSearchCV algorithm in the Python scikit-learn package, with 10 repeats of 5-fold cross-validation. The performance of the model was assessed using the receiver operating characteristic (ROC) and the area under the curve (AUC) for the validation set results. In addition, the scale_pos_weight hyperparameter was used to increase the weight of the minority class in order to avoid data imbalance issues.

The discrimination ability of the XGBoost model was quantified with the area under the receiving operating characteristic curve (AUC), sensitivity, specificity, and F1-score.

### SHAP analysis

A Shapley additive explanation (SHAP) interaction values analysis was performed for both global and local explainability, with the goal to provide a human-understandable explanation for the decisions made by the ML model.^20^ SHAP analysis is a model-agnostic methodology based on mathematical game theory. Here, the predictor variables are thought of as “players” in a cooperative game in which the goal is to obtain a reliable prediction for a single observation. Each predictor variable receives a “payout” proportional to its contribution considering all potential combinations of the predictor variables. In particular, for a specific predictor variable, the SHAP value considers the difference in the models’ predictions by including and excluding that predictor for all possible combinations of predictors. This allows to capture the average marginal contribution of each input parameter and to explain the prediction of any ML model.

In the SHAP framework, the Shapley values are output as log-odds contributions. However, the outputs in the log-odds space are difficult to interpret, so it is preferable to convert them straight to probabilities. This operation can be performed using the *model_output = ‘probability’* option, able to transform the SHAP values to probabilities directly using the DeepSHAP re-scaler.

All statistical analysis, including ML training and testing and SHAP analysis, was performed using R software (Version 3.6.1, R Foundation for Statistical Computing) and Python 3.8 (Python Software Foundation, OR, USA) packages scikit-learn, xgboost, imblearn, tabgan, and shap.

### Ethical statement

All the study procedures were compliant with the 2021 WHO guidance on ethics and governance of artificial intelligence for health.^27^

## Results

Fifty-six patients with a total of 80 lung lesions were included in this analysis. Of all lesions, 49 (61.3%) had complete response.

### Radiomics features and selection

Totally, 107 radiomics features were extracted from each lesion. CCC was 0.97 ± 0.14 overall on average. Shape features exhibited the highest score of 0.98 ± 0.08, indicating a very high interobserver reproducibility. For the texture and intensity features, the mean CCCs was ≥ 0.89.

Following data pre-processing, we performed a LASSO regression on all the considered potential predictive variables, aiming to reduce the dimensionality of the original feature space. Of the original variables, the LASSO regression algorithm selected only 9 input variables (0 clinical, 1 dosimetric and 8 radiomics) having non-zero coefficient associated with the complete response, that were therefore used for ML modelling.

### Evaluation of XGBoost model performance

**Figure 2.**
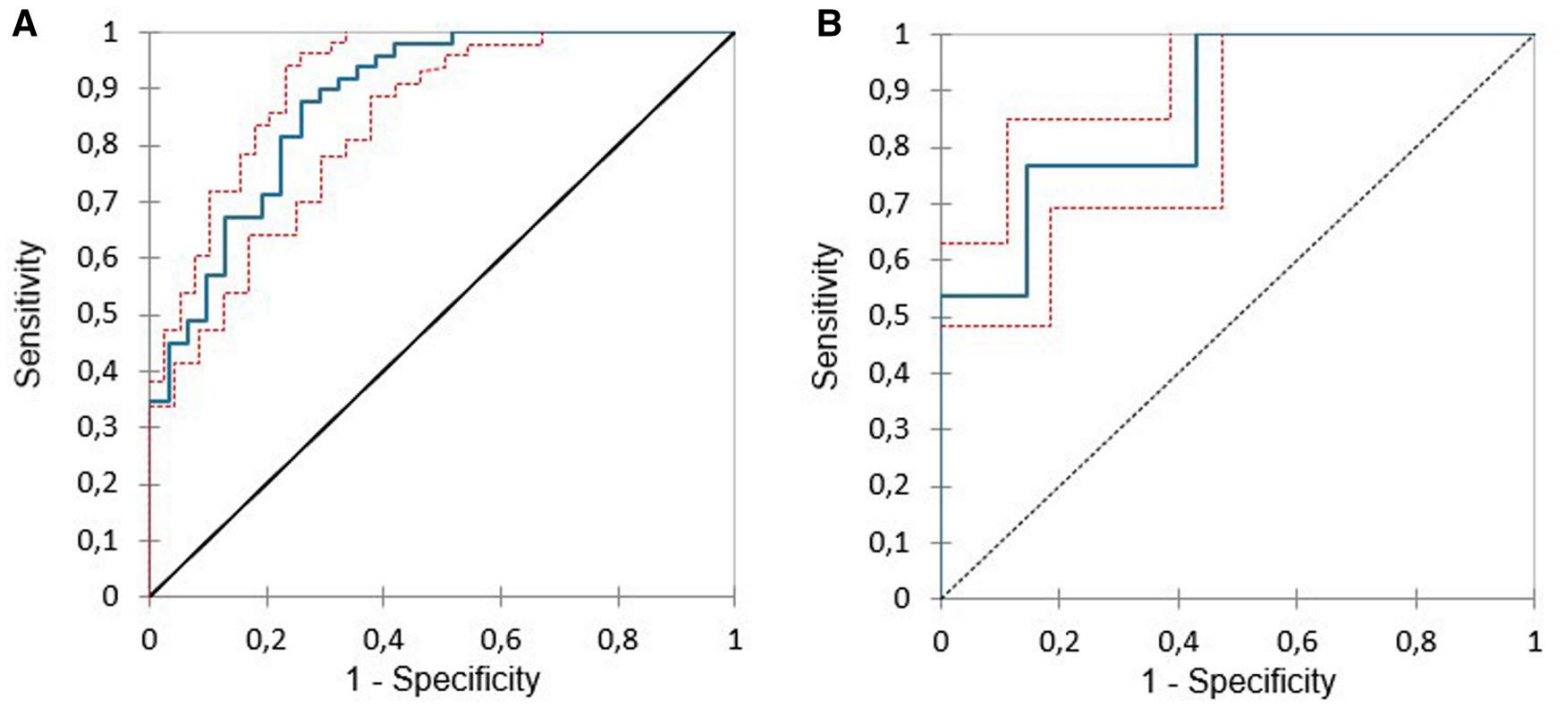
ROC curve analysis of the (A) training and (B) validation datasets.

The XGBoost model reported an accuracy, precision, recall, and F1-score equal to 0.800, 0.813, 0.923, and 0.967, respectively. Figure 2 shows the ROC curves for the XGBoost model. In the training and validation cohort, the AUC was 0.897 (95% CI 0.860-0.935) and 0.864 (95% CI 0.803-0.924), respectively.

### SHAP analysis

**Figure 3.**
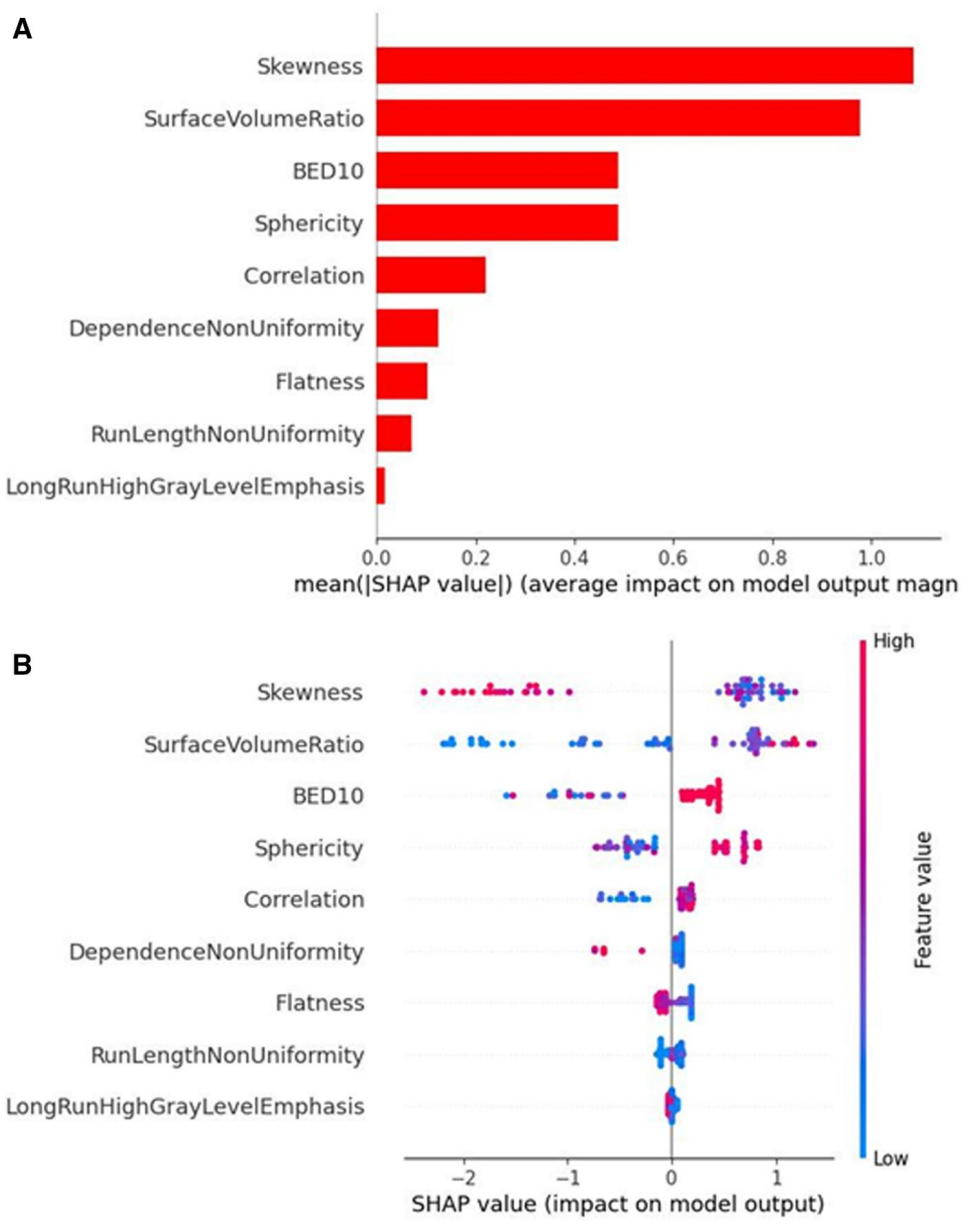
SHAP summary plot for the 9 features contributing to the XGBoost model. (A) SHAP feature importance measured as the mean absolute Shapley values. The plot depicts the importance of each covariate in the development of the final predictive model. (B) The attributes of the features in the model. The position on the y-axis is determined by the feature and on the x-axis by the Shapley value. The colour represents the value of the feature from low (blue) to high (red). Abbreviations: BED = biological equivalent dose; SHAP = Shapley additive explanations.

A global explanation of the results is reported in Figure 3. The overall contribution of the 9 significant features to the prediction of complete response is displayed in Figure 3A. Skewness, surface-to-volume ratio, sphericity, and BED10 were found to be the first 4 input variables having a significant influence on the model’s performance to predict complete response. Figure 3B shows the SHAP beeswarm plot, providing a deeper explanation of how each variable value contribute to the outcome of interest. In this graph, high and low chances of complete response are on the positive side on the x-axis (positive SHAP values) and negative side (negative SHAP values), respectively. In particular, for the first 4 features, the figure shows that higher chance of complete response is associated with lower values of the skewness and higher values of surface-to-volume ratio, BED10 and sphericity.

**Figure 4.**
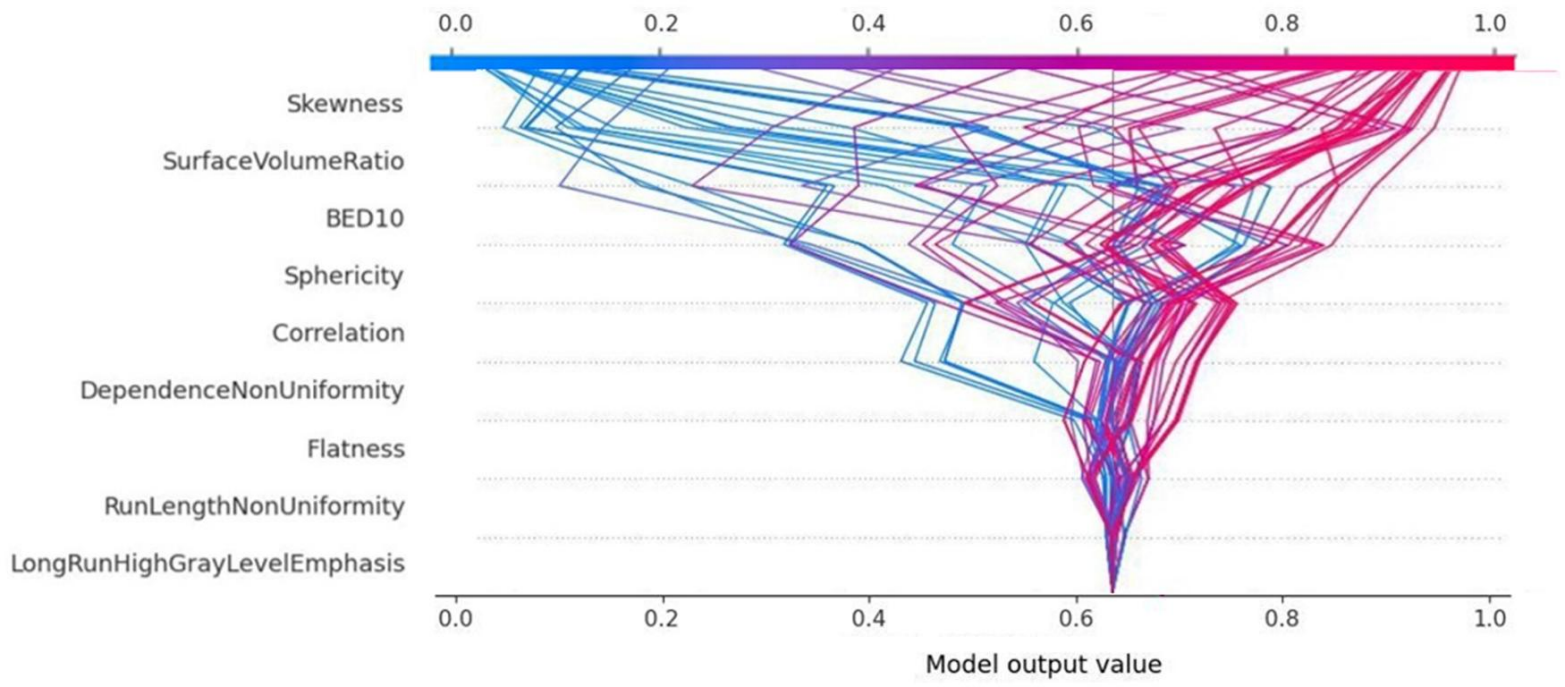
SHAP decision plots showing the prediction of survival for all patients. Abbreviation: BED = biological equivalent dose.

Figure 4 shows the global SHAP decision plot that help to understand the model decisions by mapping the cumulative SHAP values for each prediction. The x-axis represents the model’s output, that is, the probability of complete response after SBRT, after a transformation of the units from log odds to probabilities. The plot is centred on the x-axis at the “expected value.” The model’s features are listed on the y-axis in decreasing order of significance. The predicted value of each observation is represented by a coloured line. The SHAP values for every feature are added to the base value of the model, working from the bottom to the top of the plot. This illustrates how each feature affects the overall prediction and how the prediction varies during the decision process.

**Figure 5.**
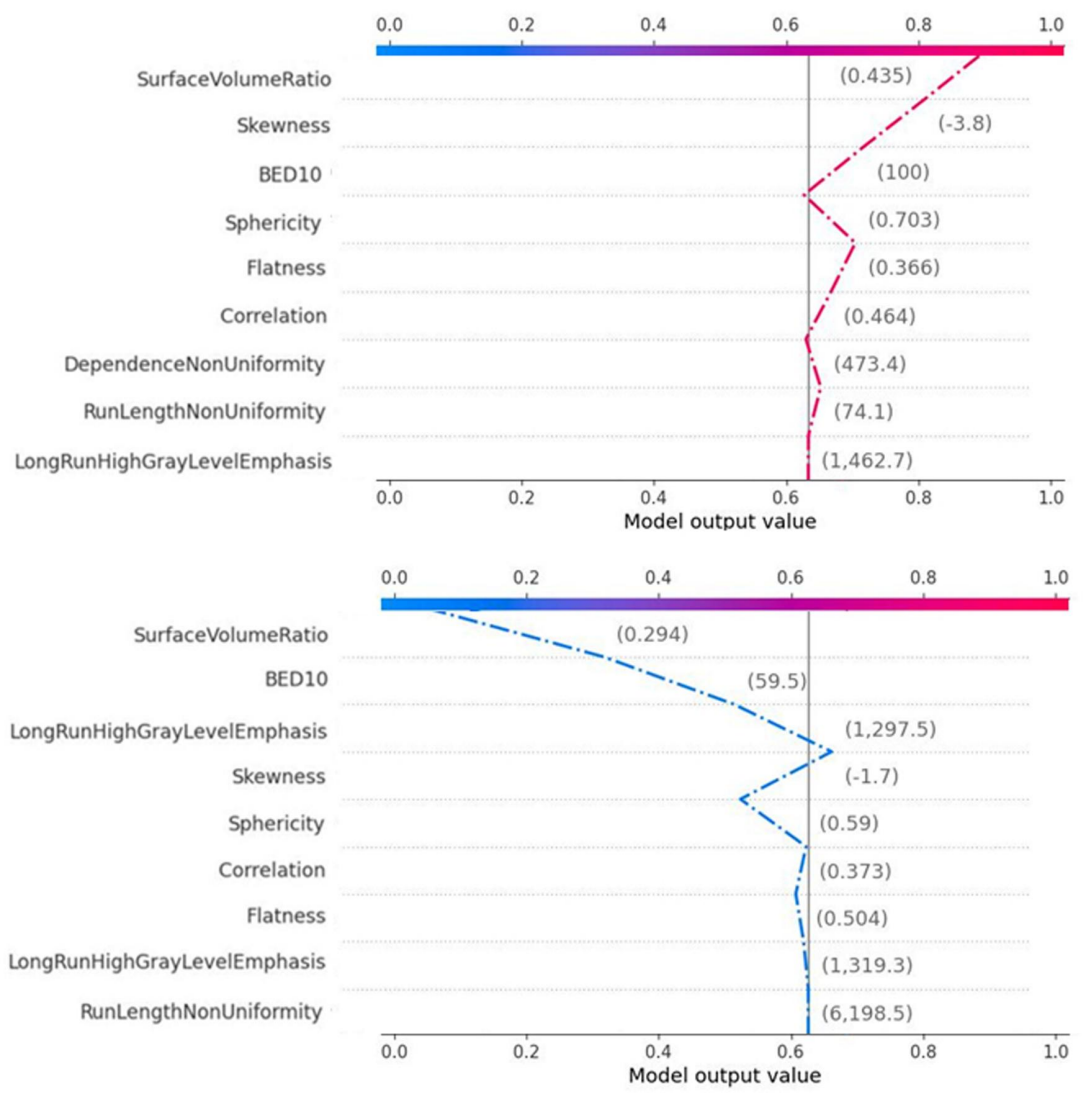
Prediction of high and low chance of complete response for 2 representative patients. The x-axis represents the probability of complete response. Red lines push the probability of complete response towards the right, that is, high chance of complete response, while the blue ones push the probability towards the left, that is, low chance of complete response. The decision path tended to make drastic turns at feature with high importance, allowing clinicians to interpret the impact of covariates on outcome. Abbreviation: BED = biological equivalent dose.

With respect to local explainability, 2 representative lesions with high and low chance of complete response are shown in Figure 5. On an individual basis, these 2 figures provide a wealth of information about the model’s decision-making process, and they may be very helpful in assisting clinicians in quickly identifying the key components of the model. The values of the input features for every patient are indicated by the numbers close to the plot. The figure is very informative at showing how the model arrived at its decision and they may be very useful in helping the clinicians to identify the major features with high decision power in the model on individual level. From Figure 5A and B, the model’s predictive probability values for complete response were 0.89 and 0.08 for the 2 patients, respectively.

**Figure 6.**
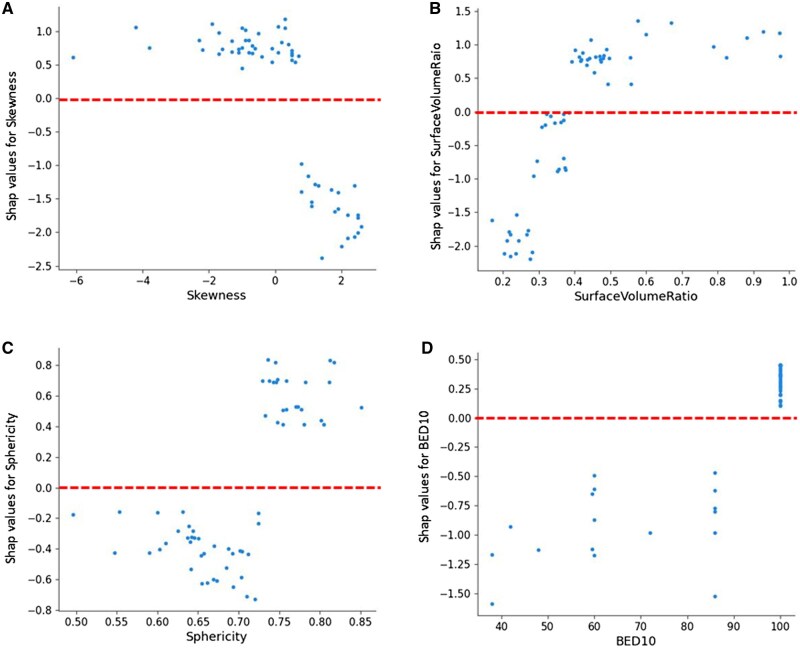
SHAP dependence plot for the first 4 features contributing to the XGBoost model: (A) skewness, (B) surface-to-volume ratio, (C) sphericity, and (D) BED10. The red dotted lines illustrate the thresholds for values driving the model towards the complete response. Abbreviation: BED = biological equivalent dose.

Using the SHAP values, it is also possible to obtain the so-called dependence plots, which display the risk of an outcome as a function of a single independent feature. Each point in this plot with a positive SHAP value corresponds to a higher probability of a complete response. Figure 6 shows the dependence plots for the first 4 variables in order of importance, that is, the skewness, surface-to-volume ratio, sphericity, and BED10. The values of skewness have an inverse proportional relationship with the chance of complete response, while the model captured a direct relationship for SVR and sphericity. The SHAP values that cross the zero values may provide a useful tool for the identification of relevant thresholds of these covariates. For instance, low values of skewness yield SHAP values greater than zero, indicating an increased chance of complete response for these patients, and an optimal threshold was identified at < 0.5. Similarly, a threshold value has been identified for surface-to-volume ratio > 0.39. For the dose, only BED10 values equal to 100 Gy have a SHAP value greater than 0.

From a clinical perspective, a more comprehensive interpretation of these findings is intriguing and will be discussed in more detail in the discussion section.

## Discussion

Over the past few years, the complete treatment response after SBRT has been strongly associated with patient’s prognosis, underlying its relevance in the oligometastatic setting. Franceschini et al^15^ assessed the impact of local response on overall survival in a large group of 358 patients who received SBRT treatments for oligometastatic disease from various solid tumours. A strong evidence of the influence of local response on outcomes was presented, with an overall survival rate at 24 months of 81.4%, 53.6%, and 38.6% in patients with complete response, PR, or stable disease, respectively. Similarly, Macchia et al^28^ presented a large real-world multicentric dataset of 846 ovarian, uterine, and cervical oligometastatic lesions treated with SBRT exploring efficacy and clinical outcomes. The authors reported a significant impact of complete response on the overall 2-year actuarial local control (91.5% for complete response vs 52.5% for incomplete response). However, despite the wide implementation of SBRT in patients with oligometastic cancer, the adoption of SBRT instead of other treatments is still at the clinician’s discretion, based on patient’s features and comorbidities. Thus, the ability to predict the radiological response before the start of an SBRT treatment may be essential for the choice of the optimal treatment modality.

At both cellular and histological levels, lung cancer is a very heterogeneous disease. As a result, studies focused only on the clinical features may be unable to fully convey the heterogeneity of lung lesions. Nowadays, it is established that tumours with irregularly spiculated edges are indicative of more infiltrating tumours, and that tumours with high heterogeneity should be regarded as more aggressive.^29^ From this perspective, radiomics’ capacity to characterize histology, genetic footprint, and intratumoural heterogeneity constitutes a promising reliable tool to provide trustworthy predictions regarding cancer outcomes.

In this study, based on radiomics analysis, we developed an explainable ML-based model to predict the treatment response of lung lesions treated with SBRT curative intent. The XGBoost model demonstrated optimal classification performances (AUC values of 0.864 in the validation cohort), and therefore, it was considered able to ascertain the probability of complete treatment response of lung lesions before receiving SBRT. Meanwhile, we demonstrated that the visualization of domain-specific feature importance provided by an explainability approach based on SHAP values can highlight the salient features of the model and can be readily understood by clinicians. Therefore, clinicians may better understand the role of specific patient or lesion characteristics in predicting the clinical outcome, then possibly guide and reinforce treatment approaches.^30^

The results of SHAP analysis, as presented in Figures 2 and 3, well explain the impact of the selected variables on complete treatment response. Interestingly, all clinical prognostic factors, such as age, gender, histological type, tumour stage, or patient’s performance status, were excluded from the model. Aside from the BED10, only radiomics features emerged as strong predictors for complete response. In particular, for the first 5 variables in order of importance, low values of the skewness and elongation, and high values of SVR, BED10, and correlation drive the model predictions towards high chance of complete response. These findings can be better understood on the basis of recent literature, summarized in Table 1.

**Table 1.** Overview of selected studies analysing the skewness, sphericity, and surface-to-volume ratio radiomics features in lung cancer.

First author, year	Reference	Aim	Methods	Results/Conclusions
Bousabarah et al. (2019)	^31^	To predict radiation-induced lung injury and outcome in NSCLC patients treated with robotic stereotactic body radiation therapy	110 patients with primary stage I/IIa NSCLC analysed for LC, DFS, OS, and local lung injury. LASSO algorithm used for features selection and continuous and dichotomous risk scores for each endpoint.	Skewness as one of the radiomic feature predicting DFS and OS and the development of local lung fibrosis in early-stage NSCLC patients treated with stereotactic radiotherapy
Coroller et al. (2016)	^32^	To predict pathological response after neoadjuvant chemoradiation in patients with locally advanced NSCLC	127 NSCLC patients analysed with radiomics to predict pathological response.	Skewness as one of the radiomic feature predicting pathological response
Chong et al. (2014)	^33^	To correlate changes of various CT parameters after the neoadjuvant treatment in patients with lung adenocarcinoma with pathologic responses	Review of pre-operative CT images of primary tumours and surgical specimens obtained after neoadjuvant therapy from 51 patients with lung adenocarcinoma.	Skewness as one of significant predictors of pathologic response
Caruso et al. (2021)	^34^	To test CTTA ability in differentiating malignant from benign nodules	46 patients who underwent FDG PET/CT followed by CTLB were retrospectively enrolled.	In malignant lesions, CTTA showed higher skewness’ values (*P* ≤ 0.0013)
Zha et al. (2024)	^37^	To explore the potential of delta radiomics from on-treatment MR imaging to track radiation dose response in lung SBRT treatments	A retrospective study of 47 MR-guided lung SBRT treatments for 39 patients was conducted. Delta radiomics were correlated with radiation dose delivery and assessed for associations with tumour control and survival with Cox regressions.	Skewness and surface-to-volume ratio decreased with radiation dose fraction delivery. Skewness was significantly associated with LF and LRFS.
Davey et al. (2019)	^36^	To investigate if sphericity of the GTV on CT planning is an independent predictor of OS in lung cancer patients treated with standard radiotherapy	Sphericity of GTV delineation was extracted for 457 lung cancer patients. Relationships between sphericity, and common patient and tumour characteristics were investigated via correlation analysis and multivariate Cox regression to assess its prognostic value.	Sphericity is strongly associated with OS (*P* < 0.001, HR (95% CI) = 0.13 (0.04-0.41)) in univariate analysis.
He et al. (2021)	^38^	To predict the presence of MP/S component in lung adenocarcinoma using radiomics analysis	268 patients undergoing curative invasive lung adenocarcinoma resection were included. Four prediction models were built by utilizing conventional machine learning approaches fitting into radiomics analysis.	Surface-to-volume ratio emerged as a strong predictor of the presence of MP/S growth patterns in lung adenocarcinoma.
Sun et al. (2020)	^39^	To investigate the value of radiomics based on CT imaging in predicting invasive adenocarcinoma manifesting as pGGNs	395 pGGNs with histopathology-confirmed benign nodules or adenocarcinoma were enrolled. A Rad-score was constructed LASSO; multivariate logistic regression analysis was conducted.	Surface-to-volume ratio was found as strong predictor of invasive lesions.
Selvam et al. (2023)	^40^	To assess the utility of radiomics in differentiating between benign and malignant lung nodules detected on CT scans	97 patients with a final diagnosis regarding the lung nodules used to build different ML classifiers.	Surface-to-volume ratio as one of significant features to differentiate benign and malignant nodules.
Trebeschi et al. (2019)	^41^	To evaluate the role of radiomics biomarkers for immunotherapy response	1055 primary and metastatic lesions from 203 patients with advanced melanoma and NSCLC undergoing anti-PD1 therapy were analysed.	In NSCLC, responding lesions presented higher levels of spherical profiles (Surface-to-volume ratio, *P* = 0.01).
Ladwa et al. (2020)	^42^	To assess CTTA of patients likely to benefit from nivolumab	47 patients were included in CTTA analysis, used to quantify heterogeneity within the tumour before immunotherapy.	At a median follow-up of 18 months, statistical significant differences in PFS were observed when stratified by positive skewness.
Weiss et al. (2014)	^43^	To evaluate the potential of tumoural CTTA to differentiate K-ras mutant from pan-wildtype tumours and its prognostic potential using baseline pre-treatment non-contrast CT imaging in NSCLC	Tumour DNA from 48 patients with early-stage NSCLC was analysed, non-parametric Mann-Whitney test assessed the ability of the CTTA to differentiate between K-ras mutation from pan-wildtype.	Positive skewness was significantly associated with the presence of a K-ras mutation.

Abbreviations: CTLB = percutaneous CT-guided Lung Biopsy; CTTA: CT-texture analysis; DFS: disease-free survival; FDG = Fluorodeoxyglucose; GTV = gross tumour volume; HR: hazard ratio; K-ras: Kirsten rat sarcoma virus; LASSO = least absolute shrinkage and selection operator; LC: local control; LF: locoregional failure; LRFS: local recurrence-free survival; MP/S: micropapillary/solid; NSCLC: non-small cell lung cancer; OS: overall survival; PFS: progression-free survival; pGGN: pure ground glass nodule; SBRT = stereotactic body radiation therapy.

The skewness, a feature describing the shape of the intensity distribution of data, has been recently considered as a potential strong signature for outcomes in lung cancer.^31–34^^,^^37^^,^^42^^,^^43^ In particular, skewness has been reported as a predictive feature for local failure and overall survival in patients treated with SBRT,^31^ for pathological gross residual disease after chemo-radiation,^33^ for pathological NR patients after chemo-irradiation^32^ and for the identification of malignant lung nodules.^34^ Following these findings, some researchers are exploring the biological basis underlying the skewness feature that may have a specific radiological appearance. For instance, skewness has been linked with KRAS mutations in NSCLC, which are indicative of treatment resistance and poorly diagnosis of lung cancers.^35^ The surface-to-volume ratio and sphericity are the shape features with stronger association with complete response. In particular, we found that lesions showing higher values of SVR and sphericity suggest a higher probability of complete response after SBRT. This outcome was expected since it has been well documented that patients with large-volume and less-spherical tumours have a worse prognosis in terms of overall survival and locoregional control.^36^ The role of BED10 is nowadays well established in literature, from the pivotal analysis of Onishi et al^44^ showing that early-stage non-small cell lung cancers receiving a BED10 ≥ 100 Gy had improved local control.

Consistent with the aforementioned literature, our results reported that skewness, surface-to-volume ratio, sphericity, and BED10 remain crucial variables in the prediction of complete response of lung lesions following SBRT. Therefore, clinicians should be aware of the significance of these features in predicting the treatment response of lesions treated with SBRT and ablative intent.

In the wake of the numerous papers published so far,^10–18^ the present research highlights the potential of radiomics in the evaluation of tumour heterogeneity and its ability to develop imaging biomarkers to predict clinical outcomes for lung tumours after SBRT. We are conscious, nevertheless, that in an oligometastatic scenario, radiomics features are only the tip of the iceberg, providing only a partial way of for the prediction of clinical outcomes. Increased predictive capacity for ML models should be possible with the inclusion of genetic or epigenetic variables, which can be used to better understand the biology underlying the diseases. The integration of genetic and radiomics data with the dosimetric and clinical ones will present novel evidence-supporting approaches capable of characterizing cancer patients, predicting prognosis, and guiding the selection of the most effective personalized treatment. For example, Wong et al^45^ were able to identify the main patterns of tumour microRNA expression that are associated with survival, developing a microRNA classifier used to determine which oligometastasis patients would benefit more from SBRT.

A few potential limitations must be highlighted. Firstly, the usual limitations of retrospective analyses apply and the size of the patient cohort is not overly large. To enable a suitable sample size for each group, we therefore select a straightforward binary classification for the response outcome (complete response vs all other responses). To improve response stratification beyond the suggested binary classification (ie, complete response, PR, stable disease, and progressive disease), we hope to collect additional lesions in the future. We are aware that an appropriate sample size is essential for obtaining a precise and reliable outcome and that studies with inadequate samples may suffer from overfitting of data. In the present paper, we used several strategies and best practices to reduce the probability of overfitting as low as possible. Firstly, we performed an accurate internal validation using a k-fold cross validation on our dataset. This involves splitting our dataset into multiple folds, training the model on different subsets, and evaluating its performance on the remaining data. This will ensure that the model is trained on 1 subset and the hyperparameters are tuned on another, in order to increase the model generalizability across the different data splits. Secondly, we used a regularization technique that added penalty terms to the loss function to prevent the model from fitting the training data too closely. For example, the L1 regularization in Lasso algorithm adds the absolute values of the coefficients to the loss function, forcing some coefficients to become exactly zero. This choice for feature selection contributes to reduce the risk of overfitting by selecting the most relevant features and exclude irrelevant or redundant ones, therefore reducing the model complexity. Despite this approach, we cannot completely exclude the occurrence of overfitting, and this possibility represents a limitation of our study. In addition, because our study was restricted to a single institution, there may be variations in image capture, target definition, and clinical outcome evaluation between institutions. Therefore, an accurate segmentation of the lesions is crucial to ensure the robustness of the extracted features. Because a detailed sensitivity analysis has not been performed, we determined the CCC for the radiomic features extracted from 2 sets of segmentations (performed by 2 different clinicians) in order to quantify the reproducibility of the features. The remarkably high CCC values (mean 0.972) indicate a well-defined target. Lastly, the performance of our model has not be verified by an external validation from a different cohort. Based on the findings of the present study, the next intention is to validate the stability and generalizability of the results in a multicentre research.

## Conclusion

In summary, our study provides a solid proof-of-concept that a radiomics-based implementation of new powerful ML models as XGBoost may strongly enhance the accuracy required for SBRT treatment response prediction. The SHAP analysis provided an intelligible explanation of treatment response prediction, enabling the radiation oncologists to understand the decision-making process hidden in the ML algorithms and directing personalized SBRT treatments in a controlled way. An external validation of our results with an independent dataset is required to confirm the robustness and generalizability of our model.
